# Effectiveness of tailored educational content on improving nurses’ clinical performance in cardiac surgery wards: a mixed-methods study

**DOI:** 10.1038/s41598-025-08728-2

**Published:** 2025-07-02

**Authors:** Hadi Kazemi-Arpanahi, Khadijeh Moulaei, Ferdos Hadideh, Zahra Gorjian, Alireza Baghrobehbahani, Maryam Heidari, Noorollah Tahery, Mohammadsadegh Aghili Nasab, Sara Sayar, Mahsa Tafazoli, Raha Tabahfar, Zeinab Nassari, Farshid Mohammad Mousaei, Elham Mohammadi Darvishvand, Mohsen Shafiee

**Affiliations:** 1 Department of Health Information Technology, Abadan University of Medical Sciences, Abadan, Iran; 2 Health Management and Economics Research Center, Health Management Research Institute, Iran University of Medical Sciences, Tehran, Iran; 3 Department of Psychiatry Counselling, Abadan University of Medical Sciences, Abadan, Iran; 4 Department of Nursing, Abadan University of Medical Sciences, Abadan, Iran; 5 School of Nursing and Midwifery, Ahvaz Jundishapur University of Medical Sciences, Ahvaz, Iran; 6 Artificial Intelligence in Medical Sciences Research Center, Smart University of Medical Sciences, Tehran, Iran

**Keywords:** Critical care nursing, Cardiac surgical procedures, Needs assessment, Health care, Medical research

## Abstract

Cardiac surgery, a complex and high-risk medical procedure, requires specialized nursing expertise. The significance of nurses’ performance in this field is paramount due to the potential consequences of errors. Therefore, nurses in cardiac intensive care units (C-ICU) must consistently undergo essential training to minimize errors and enhance their skills. This study aimed to identify the essential educational topics for nurses working in the C-ICU, provide targeted training based on these topics, and ultimately compare the clinical performance of trained nurses with those who did not receive the training. This is a mixed method study conducted in three phases. In the first phase, the educational needs of nurses were identified through a literature review and interview with 360 nurses. Subsequently, the most important educational topics were selected using a two-round Delphi study. In the second phase, a checklist to evaluate clinical performance was designed. In the final phase, 95 nurses were randomly assigned in a 2:1 ratio to either the control group (n = 60) includes untrained nurses, and the case group (n = 35) includes nurses who have received 70 h of training. Thirty case group nurses scoring above 70 advanced to the next phase. Subsequently, both case and control groups spent 6 months in hospital activities before being assessed with a checklist designed in the second phase by eight nurses. In total, 21 educational topics were approved for teaching nurses. In all topics, the mean score of the trained group (case) was higher than that of the control group (*p* < 0.05). Furthermore, the trained group exhibited a mean total score of 883 (± 36.23), whereas the untrained group showed a mean score of 566.45 (± 39.95), indicating a statistically significant difference between the two groups (*p* < 0.001). The findings of this study indicate that the performance of trained nurses was superior to that of untrained nurses. To enhance the clinical and theoretical capabilities of nurses in critical wards, it is recommended that, in addition to general education, nurses receive specialized training tailored to the specific requirements of their respective wards.

## Introduction

Cardiovascular diseases (CVDs) are a leading cause of death globally, often resulting in complications such as heart failure and stroke. Cardiac surgeries correct various CVDs and include valve repair/replacement, aortic repair, and coronary artery bypass grafting^1^. It is common for patients undergoing cardiac surgery interventions to experience depression and stress^2–4^. In order to provide specialized care to patients in the cardiac surgery ward, hospitals that perform these surgeries should adopt a team-based approach to patient care to achieve the best possible outcomes^5^. Nurses are an essential part of the care team and must possess knowledge of proper pre-and post-operative care. They must also be skilled in intensive care delivery techniques such as maintaining hemodynamic stability, monitoring for bleeding, vital signs, and breathing patterns^6–9^. Good nursing involves providing evidence-based care that meets patient needs and ensures optimal quality^10^. In 2009, the World Health Organization (WHO) encouraged member nations to enhance nurse training, leading to the establishment of global standards that significantly improved nursing practice. The need to advance basic nursing education is driven by specific global trends, including the increasing complexity of healthcare delivery, the growing elderly population, the dramatic rise in the number of people with chronic diseases and co-morbidities, and the need to ensure more equitable access to sanitary care^11^.

All issues related to patients hospitalized in the cardiac surgery ward are sensitive and important, so it’s crucial to employ trained nurses^12^. The level of knowledge and clinical performance of nurses are crucial factors that can affect the infection control and mortality rate of patients in cardiac surgery wards^13^. Therefore, nurses need to have adequate knowledge and skills to minimize procedure complications and provide optimal care^14^. Well-trained nurses with extensive knowledge are better equipped to make critical decisions and save the patient’s life in difficult situations^15^. Research has shown that training nurses can have a significant impact on treatment outcomes, including patient recovery and mortality rates^16^. Lei et al. meta-analysis and systematic review^17^ emphasizes the positive impact of this instructional approach on educational outcomes, such as improved knowledge acquisition, enhanced professional skills, and critical elements of clinical practice. Mohamed et al.^18^, also investigated the influence of simulation-based training on the communication skills, self-efficacy, and clinical competence of nursing students during practical experience. The research disclosed that this type of training in medical programs successfully improves communication skills, self-efficacy, and clinical competence.

To the best of our knowledge, no research has been conducted on the effectiveness of a tailored educational program on the performance of nurses working in cardiac surgery wards. Only some studies have focused on simulation-based cardiopulmonary resuscitation training for fourth-year nursing students, investigating its effects on knowledge, skills, and anxiety related to cardiac auscultation^19^. Other research explores the impacts of simulation-based training on nursing students’ knowledge, confidence, clinical competence, including their proficiency in advanced cardiovascular life support^20^. Additionally, studies examine the effects of high-fidelity simulation teaching on nursing students’ knowledge, professional skills, and clinical abilities^17,21^. Despite the expanding research on simulation-based training and its beneficial effects on nursing students’ knowledge, skills, and clinical competencies, a significant gap exists concerning practicing nurses in critical wards such as the cardiac surgery ward. Most current literature has primarily focused on nursing students or wider clinical environments, overlooking the distinctive educational requirements of nurses in the specialized and high-stakes context of critical wards such as the cardiac surgery ward. To date, no rigorous investigations have systematically evaluated the effectiveness of a targeted educational program aimed at enhancing the clinical performance of nurses in these critical care wards. This gap underscores the urgent need for focused interventions and empirical studies to support the ongoing professional development of nurses operating in this vital area of healthcare^22^. Therefore, this study aims to enhance the quality of care provided by nurses in the cardiac surgery ward by improving their knowledge and skills. In this regard, we specifically aim to identify the essential educational content for nurses working in cardiac surgery wards, provide training according to these contents, and investigate the effects of such training on their clinical performance.

## Methods

### Study design and setting

This mixed-method study was conducted in 2023 in Abadan, Iran, with the aim of assessing and addressing the educational needs of nurses working in cardiac surgery intensive care units (C-ICUs). Critical care units are generally categorized into two types: internal care units, such as the Coronary Care Unit (CCU), which focus on internal cardiac conditions, and surgical care units, such as the Cardiac Surgery Intensive Care Unit (C-ICU), which manage patients undergoing cardiac surgical procedures. In this study, the educational needs of nurses in C-ICUs were identified through a comprehensive process that included a literature review, a Delphi study, and interviews with practicing nurses. Based on these findings, a structured, needs-based training program was developed. Its effectiveness was evaluated through blinded assessments using a standardized checklist, which demonstrated a significant improvement in the clinical performance of participating nurses. The study proceeded through the following steps:

#### Identify the potential educational topics needed for nurses working in the cardio surgery intensive care (C-ICU)

To identify potential training topics for nurses working in C-ICU, a comprehensive literature review was conducted using scientific databases such as Web of Science, PubMed, Scopus, and Google Scholar spanning from the beginning of the search until December 2023. The inclusion criteria were as follows: studies published in English or Persian; studies that addressed the educational needs, training programs, or required competencies of nurses working in cardiac surgery intensive care units (C-ICUs) or comparable critical care settings; and publications including original research articles, review papers, clinical guidelines, or gray literature (e.g., reports and dissertations) published up to December 2023. Additionally, studies focusing on clinical performance, skill development, or simulation-based training for nurses were included.

The exclusion criteria were as follows: studies not related to nursing education or not specific to cardiac or critical care settings; articles without full-text access; studies exclusively targeting nursing students without relevance to practicing nurses in critical care environments; and duplicate publications or those presenting overlapping data across different sources. Moreover, gray literature was reviewed until no new educational topics were identified.

Finally, as part of the process to identify the educational needs of nurses working in C-ICU, an interview was conducted with 360 nurses who work in hospitals in Khuzestan province, Iran. The nurses were selected using a convenient sampling method. In the interview, each nurse was given 10 min to express their views and opinions. The following are the eligibility criteria for nurses to participate in this phase: They should be registered nurses, must have a minimum of 3 years of work experience in a C-ICU, and must be willing to participate in the study. After completing this step, a checklist was created to list all educational topics, and remove any duplicates to ensure their uniqueness.

#### Finalize the educational topics to develop a tailored educational course for nurses working in C-ICU

Using a two-round Delphi survey, a total of 40 experts were recruited which included 30 Ph.D. nursing faculty members, and ten ICU specialists. They were asked to rate the significance of educational topics derived from the previous step using a 5-point Likert scale, where 1 represented the least important and 5 represented the most important. The study participants were required to meet certain criteria, such as having at least 3 years of work experience in C-ICU, being willing to participate, and delivering the submitted checklist along with any related scientific publications, if possible. The informed consent form and checklist were sent via email to the participants. The participants were allowed to add any additional items or suggestions beyond the preliminary checklist, and they were kept unaware of each other’s responses throughout the survey.

During the Delphi survey process, educational topics with an average value of less than 50% (mean less than 2.5) were excluded. Topics with an agreement of 50% to 75% (mean 2.5 to 3.75) were re-evaluated, and any new topics suggested by the participants were considered in the next round of the Delphi method. The educational topics were considered significant if they attained an importance value of more than 75% (mean more than 3.75) in each round of the Delphi process. The time interval between each Delphi step was 2 weeks. The same panel members who participated in the first round of Delphi were also invited to participate in the second round. In the second round of Delphi, the opinions and feedback provided by the experts in the first stage were integrated into the initial items. The criteria for accepting data items remained the same as the first round.

#### Education of nurses according to derived educational topics

In this step, a case–control study was performed that employs G-Power software to determine the required sample size. To implement random sampling in our study, we utilized a combination of EPI’s cluster strategy and systematic random sampling. First, we compiled a comprehensive list of all hospitals in the province where our research is conducted, including the number of nurses in the C-ICU in each hospital. Employing the EPI methodology, we generated an aggregated list of nurses in the heart surgery ward and performed systematic random sampling from a randomly determined starting point. The participants were selected using a systematic random sampling approach, considering the number of eligible participants and the predetermined quota for each cluster. Specifically, we formed the case–control group at a ratio of 1:2, with 35 nurses in the case group and 60 nurses in the control group, totaling 95 selected nurses. This method was chosen to achieve a power of 80% and a confidence interval (CI) of 95%. The study’s case group comprised nurses who met the following inclusion criteria: a minimum of 3 years of experience in the cardiac surgery ward, full-time attendance in the training course, holding a bachelor’s degree in nursing, and obtaining a score of over 70 in the final exam. The control group, on the other hand, consisted of nurses who also had at least 3 years of experience in the cardiac surgery ward and held a bachelor’s degree in nursing, but did not participate in the training course designed for this study. All 35 nurses in the case group satisfied the inclusion criteria and qualified for the next phase of the study.

After determining nurses’ educational needs, in this step, a sample of nurses from the case group underwent a comprehensive training program tailored to their educational needs. The training consisted of theoretical and practical sessions, which lasted for 72 h in total. During the theoretical training, the nurses were taught about cardiac surgery through 20 in-person sessions, each session lasting 3.5 h. Following that, practical training was provided using simulations in the hospital to enhance the nurses’ clinical and diagnostic skills and reduce the incidence of cardiac surgery complications. After completing the training, the nurses took a test, with a minimum passing score of 70 to advance to the next phase. Thirty nurses scored above 70 and proceeded to the next phase.

#### Developing an evaluation checklist to measure the efficacy of provided education to nurses working in C-ICU

In this step, a checklist was developed to evaluate the effectiveness of the provided training course to nurses on their clinical skills and performance. The process involves the following steps:

##### Performing a literature review to collect relevant items

This is a crucial process to compile a thorough collection of necessary skills and abilities that a specialized nurse should possess. This will enable the creation of a comprehensive tool that can be utilized to gauge the effectiveness of a customized educational program on the nurses’ performance. To determine the competencies and skills necessary for nurses working in C-ICU, a comprehensive checklist was compiled after conducting a thorough literature review. Scientific databases such as Web of Science, PubMed, Scopus, and Google Scholar were used to conduct an extensive literature review from the beginning of their records until December 2023.

##### Delphi survey to identify important items

Using a two-round Delphi survey, a group of experts, including 15 Ph.D. nursing faculty members, and five ICU specialists, was assembled to assess a checklist for cardiac surgery wards. The eligibility criteria for the participants involved having a minimum of 3 years of work experience in the specialized field of cardiac surgery wards. Additionally, the participants were required to demonstrate willingness to participate in the study and deliver the completed checklist. The checklist was shared with the panel via email, and the members were encouraged to contribute to the study by suggesting additional items and rating the importance of each item on a five-point Likert scale, with scores ranging from one to five. During the Delphi survey process, items that received an average rating of less than 50% were eliminated from the study. Items that received an agreement rating between 50 to 75% were reviewed again, and any new items suggested were taken into account. Competencies were considered significant only if they received an importance rating of over 75% in each round, indicating that they were a crucial aspect of the study.

##### Content validity of the checklist

To evaluate the content validity of a given checklist, two measures were utilized: The Content Validity Index (CVI) and the Content Validity Ratio (CVR). The CVI was used to determine the relevance of each item on the checklist to the primary aims of the study. An expert panel was requested to rate each item on a Likert scale from 1 to 4, where 1 and 4 represented irrelevance and maximum relevance, respectively. The proportion of experts who rated each item with a score of 3 or 4 was calculated and used to determine the CVI for that item. A CVI score of 0.78 or more was considered acceptable for each item^23–26^.

In addition, our research involved the calculation of the Kappa statistic, a method used to minimize potential errors in the calculation of the CVI. The Kappa formula was applied to each item on the checklist, with K = (I-CVI-PC)/(1-PC). Any item that resulted in a Kappa coefficient exceeding 0.74 was deemed excellent, while items with a lower coefficient were eliminated from the checklist. This method ensured a rigorous and reliable assessment of the checklist’s content validity.

After applying rigorous statistical analysis techniques such as Delphi, Kappa, and CVI, we assigned a CVR to each item on the checklist to determine its necessity. The panel of experts rated the items, which had already passed the filtering stages of Delphi, CVI, and Kappa, on a Likert scale ranging from 1 to 3. The formula CVR = (Ne-N/2)/(N/2) was used to calculate the CVR, where Ne is the number of experts who rated the item as essential, and N is the total number of experts. Only the items with a CVR greater than 0.51, based on the Lawshe Table and the number of experts in the panel, were considered necessary and retained^27^.

#### Comparing the impact of training on nurses’ performance in the clinical environment

During the evaluation process, eight nurse assessors performed the assessments blindly. These assessors were not aware of the inclusion or exclusion criteria of the participants. To elaborate, two groups of participants were involved in the study: one acting as the case study and the other as the control group. Both groups engaged in hospital activities for 6 months, during which an intervention was implemented and evaluated for effectiveness using a checklist. The assessment process involved trained nurses completing the checklist blindly. This meant that the nurses were unaware of the group assignments and individual participants’ status (case and control) during the evaluation. The checklist, consisting of items rated on a 5-point Likert scale by eight expert nurses, served as a tool to compare the performance of the two groups. Scores ranged from 1 to 5, where 1 indicated poor and unacceptable performance, while 5 represented perfect performance. The total score ranged from a minimum of 100 to a maximum of 1000, with a score of 600 or higher indicating good performance.

Importantly, it should be noted that none of the participating nurses in the study dropped out throughout its duration. The data was analyzed using SPSS 21.0, and the Mann–Whitney test was used to determine mean scores for trained and untrained individuals.

## Results

### Identify the potential educational topics needed for nurses working in the C-ICU

To identify educational topics, a combination of literature review and semi-structured interview methods were employed. The findings of this study are as follows:

#### Performing a literature review to collect relevant topics

A comprehensive literature review was conducted to identify relevant educational topics. A total of 26 educational topics were initially extracted from the literature, which were subsequently scrutinized to eliminate duplicity or redundancy. After careful examination, 24 educational topics were accepted that included: cardiovascular anatomy and physiology, indications for cardiac surgery, preoperative cardiac surgery nursing evaluation, type of cardiac surgery and nursing care, cardiopulmonary bypass and off-Pump coronary artery bypass, hemodynamic monitoring, knowledge about the instruments used in cardiac surgery and their usage, mechanical ventilation after cardiac surgery, pharmacologic in cardiac surgery ward, postoperative complications of cardiac surgery and nursing interventions, pain management, dysrhythmias management, fluid and electrolyte imbalances, complication after cardiac surgery and their management, Wound care, Patient safety, Radiological knowledge, General and practical nursing knowledge, knowledge of interpretation of laboratory results, homovigilance knowledge, advance life support skills, infection prevention activities, communication skills, and ability to identify mental disorders.

#### Performing the Semi-structured interview to Determine the educational needs of cardiac surgery nurses

Table 1 provides an overview of the demographic information of the interviewed nurses. Out of the total participants, 58.34% were female. The mean age of the participants was 36.34 (± 7.4), and the mean work experience was 16.32 (± 6.2) years.

**Table 1:** Demographic information of the participants in the semi-structured interview.

Variables	Frequency	Percentage
Gender		
Female	210	58.34
Male	150	41.66
Educational		
Bachler	160	44.45
Graduate	120	33.34
Post-graduate	80	22.21
Age		
25–30	39	10.84
30–35	68	18.89
35–40	105	29.17
40–45	103	28.62
> 45	45	12.48
Work experience		
3	12	3.34
3–8	45	12.48
8–13	96	26.67
13–18	102	28.34
> 18	105	29.17
Total	360	100

	Mean	SD
Age	36.4	± 7.4
Work experience	16.32	± 6.2

After conducting interviews with the invited nurses, we analyzed their educational requirements. We eliminated duplicate topics and compiled the remaining topics into an information pool. This process helped us identify 11 educational topics that require attention. These topics are as follows: cardiovascular anatomy and physiology, indications for cardiac surgery, cardiopulmonary bypass and off-pump coronary artery bypass, management of dysrhythmias, fluid and electrolyte imbalances, complications after cardiac surgery and their management, mechanical ventilation after cardiac surgery, radiology, interpretation of laboratory results, knowledge of hemovigilance, and an understanding of the instruments used in cardiac surgery and their usage.

### Finalize the educational topics to develop a tailored educational course for nurses working in C-ICU

Table 2 shows the characteristics of the participants in the Delphi phase. According to this table, most of the participants (52.5%) were women. Also, the highest frequency was related to the age group of 40–50.

**Table 2:** Characteristics of the participants in the Delphi phase.

Variables	Frequency	Percentage
Gender		
Female	21	52.5
Male	19	47.5
Educational		
PhD in nursing and nursing faculty members	30	75
ICU specialists	10	25
Age		
30–40	15	37.5
40–50	20	50
> 50	5	12.5
Work experience		
< 10	6	15
10–15	10	25
15–20	10	25
20–25	8	20
> 25	6	15
Total	40	100

	Mean	SD
Age	35.4	± 6.4
Work experience	14.32	± 5.2

Table 3 presents the findings of the Delphi technique employed to evaluate the educational topics checklist. Initially, the checklist comprised 24 items which underwent a rigorous screening process, resulting in the removal of one item in the first phase. In this phase, there items were approved, with only one item proceeding to the second phase, which was subsequently eliminated. The table offers a comprehensive overview of the subjects that need to be taught based on the Delphi calculation.

**Table 3:** Delphi phase to indentation important items.

Educational needs		Round 1 of Delphi			Round 2 of Delphi			Decision
		Agree %	Disagree %	Unsure %	Agree %	Disagree %	Unsure %	
1	Cardiovascular anatomy and physiology	100%	0	0				✓
2	Indications for cardiac surgery	100%	0	0				✓
3	Preoperative cardiac surgery nursing evaluation	100%	0	0				✓
4	Type of cardiac surgery and nursing care	100%	0	0				✓
5	Cardiopulmonary bypass and off-pump coronary artery bypass	90%	0	10%				✓
6	Hemodynamic monitoring	100%	0	0				✓
7	Knowledge about the instruments used in cardiac surgery and their usage	97.5%	2.5%	0				✓
8	Mechanical ventilation after cardiac surgery	100%	0	0				✓
9	Pharmacologic in cardiac surgery ward	100%	0	0				✓
10	Postoperative complications of cardiac surgery and nursing interventions	100%	0	0				✓
11	Pain management	87.5%	10%	2.5%				✓
12	Dysrhythmias management	100%	0	0				✓
13	Fluid and electrolyte imbalances	100%	0	0				✓
14	Complication after cardiac surgery and their management	100%	0	0				✓
15	Wound care	80%	20%	0				✓
16	Patient safety	100%	0	0				✓
17	Radiological knowledge	87.5%	10%	2.5%				✓
18	General and practical nursing knowledge	65%	35%	0	60%	40%	0	×
19	Knowledge of interpretation of laboratory results	80%	20%	0				✓
20	Homovigilance knowledge	100%	0	0				✓
21	Advance life support skills	100%	0	0				✓
22	Infection prevention activities	95%	0	5%				✓

*Assessment in first and second round Delphi, ×: Final exclusion and ✓: Final Acceptance.

### Education of nurses according to derived educational topics

The training course was attended by 35 participants, out of which 30 individuals completed the course and passed, while 5 people failed to pass the training course. Following the course completion, the clinical performance of these 30 trained nurses was compared with that of the 60 untrained individuals.

### Developing an evaluation checklist to measure the efficacy of provided education to nurses working in C-ICU

#### Performing a literature review to collect relevant items

A comprehensive literature review was conducted to identify relevant competencies. A total of 350 competencies were initially extracted from the literature, which were subsequently scrutinized to eliminate duplicity or redundancy.

#### Delphi survey to identify important items

A panel of experts, which consisted of 15 nursing faculty members in intensive care, and five ICU specialists, was provided with an initial checklist containing 350 items. The participants were 60% female and 40% male, with a mean age of 32.65 ± 6.3 SD and a mean work experience of 17.25 ± 5.2 SD at the hospital. Table 4 presents the results of the Delphi process used to evaluate the educational topics in the checklist. Out of the initial 350 items, 250 were selected, while 100 were removed.

**Table 4:** Delphi phase, and calculating CVI, kappa, and CVR to identify items for mechanical ventilation after cardiac surgery topic.

Main topic: mechanical ventilation after cardiac surgery		Round 1 of Delphi			Round 2 of Delphi			CVI	Kappa	CVR	Decision
		Agree %	Disagree %	Unsure %	Agree %	Disagree %	Unsure %				
1	Ability to set up the ventilator according to the patient’s condition	100%	0	0				1	1	1	Kept
2	Ability to identify predictors of prolonged mechanical ventilation	95%	5%	0				0.95	0.999	0.9	Kept
3	Prevention of complications of prolonged ventilation	100%	0	0				1	1	1	Kept
4	Ability to identify and manage complications of prolonged ventilation	90%	10%	0				0.9	0.988	0.8	Kept
5	Ability to assessment of preoperative predictors for weaning	90%	0	10%				0.9	0.988	0.8	Kept
6	Ability to assessment of postoperative predictors for weaning	100%	0	0				1	1	1	Kept
7	Evaluation of pulmonary mechanics: Evaluation of certain parameters is suggested to evaluate patient readiness to wean—namely, vital capacity, minute ventilation (or volume), respiratory rate, tidal volume, and negative inspiratory pressure	95%	5%	0				0.95	0.91	0.9	Kept
8	If, a postoperative patient requires prolonged ventilator support for more than several days, so, nurse has the nurse has the ability to assessment complications such as atelectasis, left ventricular failure, pleural effusion, and phrenic nerve Injury	100%	0	0				1	1	1	Kept
9	Ability to assessment of arterial blood gas and ability to diagnose acid–base disorders and their causes	100%	0	0				1	1	1	Kept
10	Ability to perform correct nursing intervention to correct acid–base disorders	100%	0	0				1	1	1	Kept
11	Ability to perform initial postoperative ventilator settings	85%	10%	5%				0.85	0.978	0.7	Kept
12	Ability patient monitoring	100%	0	0				1	1	1	Kept
13	Ability to identify extubation criteria	100%	0	0				1	1	1	Kept
14	Ability to perform post-extubation care	100%	0	0				1	1	1	Kept

#### Content validity of the checklist

CVI of each item was measured. Then, 30 items were removed. Kappa coefficient of each checklist item was measured and 15 items were removed. So, after calculating CVR of the checklist items, the other five items were removed and the final checklist was developed with 200 items (Table 4).

### Comparing the impact of training on nurses’ performance in the clinical environment

Table 5 shows the demographic information of the participants. According to this table, 50% of participants were female. The mean age in the case group was 34.90 (± 2.35) and was 35.50 (± 2.78) in the control group.

**Table 5:** descriptive demographic information in two group.

Variables	Trained people (case)	Untrained people (control)
Gender		
Male	15 (50%)	30 (50%)
Female	15 (50%)	30 (50%)
Age	34.90 (± 2.35)	35.50 (± 2.78)
Work experience	12.60 (± 2.55)	13.55 (± 2.83)

Table 6 compares the mean score obtained from the checklist, age and work experience in the two groups. 100% of trained people had good clinical performance.

**Table 6:** Descriptive data in two group based on gender.

Mean score	Trained people		Untrained people	
	Male	Female	Male	Female
Total core	877.93 (± 21.50)	889.53 (± 46.74)	567.30 (± 42.08)	565.60 (± 38.39)
Age	35.66 (± 2.82)	34.13 (± 1.50)	35.60 (± 2.66)	35.40 (± 2.95)
Work experience	13.53 (± 2.94)	11.66 (± 1.71)	13.60 (± 2.66)	13.50 (± 3.03)
Good performance	100%	100%	10%	23.3%

The mean score obtained in the trained group was higher than that of the untrained group and Mann–Whitney statistic was significant (Table 7).

**Table 7:** Compare mean in two groups (case and control group).

Educational topics		Case	Control	Statics	*P* value
		Mean score of trained people	Mean score of Untrained people		
1	Cardiovascular anatomy and physiology	48.86 (± 2.50)	25.83 (± 3.09)	Mann–Whitney Test	< 0.001
2	Indications for cardiac surgery	38.26 (± 2.14)	21.90 (± 2.23)	Mann–Whitney Test	< 0.001
3	Preoperative cardiac surgery nursing evaluation	47.86 (± 2.23)	25.73 (± 3.09)	Mann–Whitney Test	< 0.001
4	Type of cardiac surgery and nursing care	39.26 (± 3.14)	22.90 (± 2.23)	Mann–Whitney Test	< 0.001
5	Cardiopulmonary bypass and off-pump coronary artery bypass	18.73 (± 1.85)	13.58 (± 2.03)	Mann–Whitney Test	0.002
6	Hemodynamic monitoring	24.13 (± 1.30)	14.60 (± 0.49)	Mann–Whitney Test	< 0.001
7	Knowledge about the instruments used in cardiac surgery and their usage	18.73 (± 0.85)	14.58 (± 1.03)	Mann–Whitney Test	0.004
8	Mechanical ventilation after cardiac surgery	64.86 (± 3.69)	44.71 (± 4.70)	Mann–Whitney Test	< 0.001
9	Pharmacologic in cardiac surgery ward	47.50 (± 2.55)	28.80 (± 4.43)	Mann–Whitney Test	0.005
10	Postoperative complications of cardiac surgery and nursing interventions	46.50 (± 2.35)	29.82 (± 3.43)	Mann–Whitney Test	< 0.001
11	Pain management	24.06 (± 2.07)	13.68 (± 2.25)	Mann–Whitney Test	< 0.001
12	Dysrhythmias management	45.50 (± 1.55)	27.80 (± 2.43)	Mann–Whitney Test	< 0.001
13	Fluid and electrolyte imbalances	46.50 (± 1.65)	26.80 (± 2.33)	Mann–Whitney Test	< 0.001
14	Complication after cardiac surgery and their management	96.80 (± 3.24)	71.33 (± 5.85)	Mann–Whitney Test	< 0.001
15	Wound care	18.73 (± 1.85)	13.58 (± 2.03)	Mann–Whitney Test	0.003
16	Patient safety	25.13 (± 1.30)	15.60 (± 1.49)	Mann–Whitney Test	< 0.001
17	Radiological knowledge	17.73 (± 0.85)	12.58 (± 1.03)	Mann–Whitney Test	0.003
18	Knowledge of interpretation of laboratory results	18.83 (± 1.75)	13.78 (± 1.23)	Mann–Whitney Test	0.003
19	Hemovigilance knowledge	95.80 (± 2.24)	74.33 (± 3.85)	Mann–Whitney Test	< 0.001
20	Advance life support skills	44.63 (± 1.55)	26.80 (± 2.43)	Mann–Whitney Test	0.003
21	Infection prevention activities	48.20 (± 1.65)	24.48 (± 2.33)	Mann–Whitney Test	< 0.001
22	Total core	883 (± 36.23)	566.45 (± 39.95)	Mann–Whitney Test	< 0.001

## Discussion

This study utilizes a mixed-methods approach to identify the educational needs of cardiac surgery ward nurses and develop tailored educational programs to address these needs. The study findings have the potential to improve patient outcomes and enhance the overall quality of care in the cardiac surgery ward. This study involved extracting 21 educational titles and conducting a 72-h training course. A checklist was created to assess the impact of the training on the clinical performance of nurses. The clinical performance of nurses in the control and case groups was evaluated based on this checklist. The study observed an average performance score of 883 (± 36.23) in the case group and 566.45 (± 39.95) in the control group. The case group had a significantly higher score compared to the control group, indicating that training tailored to the educational needs of a department can have a positive impact on the clinical performance of nurses. This improvement in performance can prove beneficial in the diagnosis and treatment of patients in specialized departments. This study advanced nursing knowledge by focusing on a critical gap in the existing literature concerning the clinical education of practicing nurses within cardiac surgery units. Unlike prior studies that have predominantly examined nursing students or broader clinical environments, this investigation specifically addresses the intricacies and challenges inherent in the specialized, high-stakes context of cardiac surgical care. It underscored the necessity of customized educational programs designed to elevate nurses’ expertise and clinical effectiveness in the specialized field of cardiac surgery. It aims to systematically identify key educational components essential for nursing professionals working in cardiac surgery units, implement focused training modules based on these identified needs, and assess the resultant impact of this targeted education on both clinical competence and the quality of patient care delivered by nurses. The study provided empirical evidence indicating that implementation of structured, ward-specific training programs can enhance nursing competencies, lead to improved patient outcomes, and mitigate complications such as infections and mortality rates in cardiac surgery environments. This research underlines the importance of continuous professional development for nurses and underscores the critical role of specialized education in optimizing healthcare delivery within intricate clinical settings. One of the key strengths of this study is the application of a mixed-methods approach, which combines qualitative insights with quantitative evaluation to comprehensively identify the educational needs of nurses and assess the effectiveness of tailored training programs. This methodological integration enhances the validity and robustness of the findings by capturing both subjective perceptions and objective performance outcomes^28^. Critical care nurses in the ICU must possess advanced clinical expertise and theoretical knowledge due to the complex and critical health conditions of patients. They must be proficient in advanced assessment techniques, decision-making processes, and the use of sophisticated medical equipment to ensure optimal patient outcomes^12^.

The study’s emphasis on practicing nurses within C-ICUs represents a significant strength, particularly given the specialized nature of this clinical environment. This focus distinguishes it from prior research, which frequently concentrates on either general ICU populations or nursing students, thus providing valuable insights tailored to the complexities of C-ICUs^29^, this study specifically identifies the advanced competencies essential for the provision of cardiac surgical care, thereby addressing a significant gap in the existing literature. The formulation of the training program, grounded in a systematic assessment of educational needs, constitutes a significant advantage. This needs-driven methodology guarantees that the educational content is not only pertinent but also directly applicable to clinical practice, thereby enhancing its overall effectiveness and impact^30^. The implementation of a structured 72-h training program, coupled with a validated checklist for the assessment of nurses’ performance both pre- and post-intervention, enhances methodological rigor. This approach facilitates precise monitoring of improvement metrics^31^. Several studies have highlighted the significant benefits of nurse training, supporting the findings of our study. Lei et al. study^17^ not only emphasizes but robustly asserts that training programs constitute a pivotal factor in enhancing the clinical performance of nurses. Through the meticulous addressing of knowledge gaps and the cultivation of continuous improvements in patient care, these training initiatives significantly contribute to the overall competence of nursing professionals. In a parallel vein, Hegland et al.^29^, inquiry delves into the realm of empathy-focused training, uncovering its affirmative impact on the knowledge and practical application of empathic communication behaviors among neonatal intensive care unit (NICU) nurses. According to Goodarzi et al., nearly half of the nurses demonstrated a comprehensive understanding of infection control and prevention via proper hand hygiene techniques, while the remaining half did not perform as proficiently. The study highlighted a distinct correlation between training and performance, indicating that consistent training for nurses could significantly enhance both their knowledge and performance in this critical area^30^.

The pronounced disparity in performance metrics between the intervention and control cohorts offers compelling empirical evidence for the efficacy of the targeted training program. This underscores that tailored, context-relevant educational interventions can significantly enhance nursing competencies, minimize clinical errors, and potentially improve patient outcomes in specialized healthcare environments^31^.

Moreover, Malmström et al.'s insightful research^32^ sheds light on the remarkable enhancement of self-evaluated proficiency in neonatal resuscitation among physicians, nurses, and midwives. Their findings highlight a noteworthy improvement subsequent to a comprehensive training course that encompassed simulation-based team training and video-supported debriefing. The amalgamated consensus derived from these studies not only reaffirms but amplifies our own findings, accentuating the pivotal role of nurse training in elevating healthcare quality and, by extension, improving patient outcomes.

Considering the cumulative evidence from these studies, it is prudent to recommend a strategic and comprehensive approach to nurse training programs. Initiatives should be tailored to address specific areas such as clinical performance, empathy development, and proficiency in critical procedures like neonatal resuscitation. Incorporating simulation-based team training, as demonstrated by Malmström et al., could be particularly beneficial. Additionally, fostering a culture of continuous learning and professional development within healthcare institutions is crucial. Regular assessments and updates to training programs based on the evolving needs of the healthcare landscape will ensure that nurses remain well-equipped to provide high-quality care. Lastly, creating a supportive environment that encourages empathy and effective communication should be an integral part of any nurse training program. These suggestions aim to enhance the impact of nurse training on overall healthcare quality and patient outcomes.

On the other hand, several studies have also indicated that the absence of nurse training can lead to challenges. Bankanie et al. also have highlighted the lack of essential knowledge among nurses in certain areas owing to multiple factors such as job dissatisfaction, inadequate educational guidelines, and lack of motivation. The authors suggest that addressing these challenges could bring about better outcomes. Hence, it is crucial to improve educational guidelines and investigate the factors that contribute to nurses’ lack of motivation and job satisfaction. These measures could significantly enhance the quality of care and ultimately lead to improved patient outcomes in this field^33^. Rezaei et al. examined the quality of the suctioning procedure and ICU nurses’ level of knowledge regarding the care before, during, and after it. The results showed 19.2%, 65.4%, and 15.4% of nurses had poor, moderate, and good performance, respectively. However, none of the nurses had excellent and flawless performance, indicating a gap between nurses’ knowledge and clinical performance. Thus, nurses need to be trained and continuously update their knowledge^34^. Recent studies conducted by Meng’anyia et al. and Sowan et al. reveal that nurses lack sufficient knowledge in the field of medical device maintenance and require both theoretical and practical training^35,36^. According to Tarhan et al.'s study, only 55.6% of nurses demonstrated adequate proficiency in chest drainage care and management of complications. The remaining 44.4% of nurses lacked sufficient knowledge and skills, indicating the need for targeted training programs to improve their performance^37^. Abuesheisheh and colleagues’ research revealed that the vast majority of participants exhibited inadequate knowledge regarding chest drainage care and related complications. As a result, it is imperative to implement a continuous training program for nurses to enhance their proficiency in this area^38^. After a thorough review of the literature, it has been established that regular training sessions play a crucial role in enhancing the proficiency and knowledge of nurses. This finding suggests that continuous professional development is necessary for nurses to improve their performance and ensure quality healthcare delivery^39^. In conclusion, the collective evidence from various studies underscores the critical impact of nurse training on the overall competence and proficiency of healthcare professionals.

## Limitations of the study

This study faced several limitations. First, it was conducted in a single geographic region, which limits the generalizability of the findings to other areas or healthcare settings with different practices or resources. The follow-up period of 6 months may not have been sufficient to capture the long-term effects of the training on nurses’ performance and skill retention. Additionally, the sample size was relatively small, which may not fully represent the broader population of nurses working in cardiac surgery wards. Finally, the study did not use a randomized controlled trial (RCT) design, which would have provided stronger evidence for the causal relationship between the training and the observed performance improvements.

To overcome these limitations, future studies should aim for a larger and more diverse sample, potentially including multiple geographic regions and healthcare settings to enhance the external validity of the findings. A longer follow-up period could provide more insight into the long-term effectiveness of the training program. Implementing a randomized controlled trial design could strengthen the internal validity of the study and provide more robust evidence of the causal link between training and performance improvements. Moreover, to reduce self-selection bias, random assignment or efforts to encourage a broader range of participants could be employed.

## Strengths of the study

This study exhibited several notable strengths that contribute to the robustness of the findings. The mixed-methods design, combining both qualitative and quantitative data, provided a comprehensive understanding of the educational needs and the impact of the tailored training on clinical performance. The educational content was specifically designed based on the actual needs of the nurses, ensuring that the training was relevant and targeted. The validation process for the evaluation checklist was rigorous, which increased the reliability of the clinical performance assessment. Additionally, the implementation of the training program in a real clinical setting offered practical insights into its effectiveness. The use of blinded evaluators helped minimize potential biases in the assessment of nurse performance, further enhancing the credibility of the results.

## Conclusions

This study delved into assessing the impact of a tailored educational program on the performance of nurses within cardiac surgery wards. The findings unequivocally revealed that the performance of those who underwent specialized training surpassed that of their untrained counterparts. The implications derived from this research underscore the critical importance of not only general education but also specialized training for nurses in critical care settings. To enhance both clinical and theoretical proficiency in specialized wards, it is imperative to integrate targeted education and training into the nurse’s professional development. This specialized education, tailored to the unique demands of cardiac surgery wards, proved instrumental in elevating the overall performance of trained nurses. Recognizing the dynamic nature of healthcare, it is recommended that, upon entering the workforce, nurses receive ongoing training aligned with the specific educational needs of their designated ward. Thus, a comprehensive approach to education ensures that nurses are well-equipped to navigate the complexities of their respective fields, ultimately fostering a healthcare environment characterized by excellence and enhanced patient care.

## Data Availability

The datasets used and/or analyzed during the current study are available from the corresponding author on reasonable request.
